# Development and validation of a clinical sleep assessment tool for patients with cancer during treatment

**DOI:** 10.1016/j.apjon.2025.100733

**Published:** 2025-05-28

**Authors:** Mats Nilsson, Delmy Oliva, Bengt-Åke Andersson, Freddi Lewin, Lasse D. Jensen

**Affiliations:** a Futurum, Academy of Health and Care, Region Jönköping County, Jönköping, Sweden; b Department of Biomedical and Clinical Sciences, Linköping University, Linköping, Sweden; c Department of Oncology, Ryhov County Hospital, Jönköping, Sweden; d Department of Clinical Diagnostics, School of Health and Welfare, Jönköping University, Jönköping, Sweden; e Department of Medicine, Health and Caring Science, Division of Diagnostics and Specialist Medicine, Unit of Cardiovascular Medicine, Linköping University, Linköping, Sweden

**Keywords:** Cancer, Chemotherapy, Sleep, Sleep disruption, Circadian rhythm

## Abstract

**Objective:**

Sleep disruption is common among patients with cancer, negatively impacting treatment outcomes, survival, and quality of life. However, it is often overlooked in cancer care. This study aimed to explore shared characteristics of sleep disruption in patients with cancer to facilitate simple and accurate identification in routine clinical practice. A secondary aim was to identify potential biomarkers in urine, serum, or leukocytes associated with sleep disruption before and/or after oncological therapy.

**Methods:**

Ninety cancer patients scheduled for either adjuvant or palliative oncological therapy at Ryhov County Hospital, Jönköping, Sweden, were consecutively enrolled. Of these, 72 completed all questionnaires and provided urine and blood samples at both baseline and three-month follow-up. Data were collected using the 12-item Medical Outcomes Study Sleep Scale (MOS-SS) and the 30-item European Organization for Research and Treatment of Cancer Quality of Life Questionnaire (EORTC QLQ-C30). Biomarker analysis was conducted on urine and blood samples, and data were analyzed using ordinal factor and Rasch modeling.

**Results:**

Two distinct factors—Sleep Quality (SQ) and Daytime Sleepiness (DTS)—emerged from the MOS-SS, effectively capturing key aspects of sleep disruption. Both SQ and DTS were strongly associated with sleep-related impairments identified via the EORTC QLQ-C30 and clinical history, but showed no correlation with urinary melatonin or cortisol, serum inflammatory cytokines, or Bmal1 and Per2 gene expression in blood leukocytes. Neither SQ nor DTS was significantly influenced by patient age, body mass index (BMI), or oncological therapy. However, women reported significantly lower DTS compared to men (*P* ​< ​0.05), while SQ remained unaffected by sex. A simplified scoring tool for SQ and DTS was developed for practical use in clinical oncology settings.

**Conclusions:**

This study identifies SQ and DTS as robust measures of sleep quality and daytime sleepiness in cancer patients. These new factors derived from the MOS-SS can support the early detection and management of sleep disruption in routine oncological care.

## Introduction

Sleep disruption is a common co-morbidity in patients with cancer affecting approximately half of the patients.^1^ Many sleep quality improving interventions have been developed and tested within oncological care regimens, showing clear benefits both on quality of life as well as treatment outcomes and cancer-specific survival.^2^, ^3^, ^4^ Diagnosis of sleep disruption is, however, often neglected in patient care, possibly due to the lack of clinically manageable and validated approaches that would allow for individualised measures. The gold standard for diagnosis of sleep disruption is though polysomnography.^5^ This is, however, a complicated technique requiring specialized infrastructure (sleep labs). To facilitate diagnosis of sleep disruption it has been proposed to measure the levels of biomarkers with a known circadian rhythm in the cells (such as Bmal1 and Per2),^6^ blood (such as interleukin 6 [IL-6] and C-reactive protein [CRP]),^7^^,^^8^ or urine (such as melatonin metabolites or cortisol).^6^^,^^9^ As these biomarkers, however, may also be changed in diseases such as cancer, it is unclear if they have value as diagnostic tools for assessment of sleep disruption in patients. As an alternative, simpler methods such as accelerometers or subjective patient-reported outcome instruments such as the Pittsburgh Sleep Quality Index, Epworth Sleepiness Scale or the Medical Outcomes Study – Sleep Scale (MOS-SS) are commonly used.^10^ Among these, the sleep scales provide quantifiable readouts of the experienced sleep quality,^11^^,^^12^ which have been suggested as a more accurate determinant of pathological sleep disruption compared to physical activity measurements by accelerometers.^13^ These sleep scales have, however, not been implemented in routine clinical practice possibly because of the complexity of analysing the responses.^14^ For example, an analysis of the answers to the 12-item MOS-SS results in seven different, partially overlapping, sub-scales.^15^ Clinically, it is difficult to interpret all these sub-scales and arrive at a diagnosis of whether the patient is affected by sleep disruption or not. On the other hand, sleep disruption is a complex phenotype that cannot be accurately diagnosed in a broad and heterogeneous patient population by simply asking the patient if he or she sleeps well.^16^ Therefore, an intuitive, yet robust, methodology that provides clinically valuable information related to sleep disruption in patients with cancer is urgently needed.

Previous reports have linked sleep deprivation among patients with cancer to nocturnal urination, anxiety, illness-related concerns, and side effects like pain and nausea/vomiting induced by oncological treatments.^17^ Furthermore, patients experiencing deteriorating sleep quality during oncological treatment had higher serum levels of pro-inflammatory cytokines.^18^ Oncological therapy may, however, affect sleep differently in patients depending on whether or not a medical benefit from the therapy is achieved, and on the severity of treatment-associated side-effects.^19^ On the other hand, high quality sleep was associated with improved overall survival in colorectal patients with cancer on adjuvant chemotherapy.^20^ These findings illustrate the complexity of the interactions between sleep quality and oncological treatments and highlights the importance of tools for monitoring sleep quality in patients with cancer.

The aim of this study was therefore to determine if the MOS-SS could be improved to provide an intuitive and accurate measurement of sleep disruption in patients with cancer that would be implementable within routine clinical practise. Further, we aimed to identify single urinary, serum or gene-expression biomarkers associated with sleep disruption in this patient group before and/or after oncological therapy.

## Methods

### Patients

The study was designed as a longitudinal observational study. Between 2017 and 2018, 90 patients receiving systemic, oncological therapy at the Oncology Clinic, Ryhov County Hospital in Jönköping, Sweden were enrolled in this study. The enrolled patients were diagnosed with various types of cancer, including gastrointestinal, urothelial, breast, brain, and tonsillar cancers. Patients’ inclusion criteria were 1) planned adjuvant or palliative oncological therapy, 2) Eastern Cooperative Oncology Group (ECOG) Performance status of 0 or 1 at inclusion, and 3) Sufficient understanding of Swedish to comprehend the information provided. The exclusion criterion included 1) patients that did not understand Swedish, or 2) performance status was higher than one (e.g., patients that are not capable of carrying out any work activities other than self-care). An oncology nurse provided both oral and written information about the study, and patients had 30 minutes or until their next appointment to decide on their participation. Informed consent was given in writing by all included patients prior to the first day of the study.

### Patient reported outcomes instruments and clinical data collection

Data for this study were collected in three ways to estimate sleep disruption: (a) the MOS-SS (Swedish version, MOS 12-item Sleep Scale-Revised),^15^ (b) semi-structured interviews (anamnesis) with specific questions coupled to individualized follow-up questions when needed, and (c) the European Organization for Research and Treatment of Cancer (EORTC) quality of life 30-question (QLQ-C30) questionnaire.^21^ In addition, information from patients' medical records including demographics, the type of cancer and treatment, tumour stage classification (tumor-node-metastasis [TNM]),^22^ and the ECOG performance status.^23^ These details were reported in a case report form (CRF) were also used in the study (Supplementary Table S1). For all included patients, the MOS-SS, EORTC QLQ-C30 urine, and blood samples were collected on the first day of treatment and after three months at the first treatment follow-up.

The MOS-SS evaluates the respondents sleep habits during the last four weeks. For items 3 to 12 (item 1 and 2 not included in the scoring process) the response on the items varies from “All of the time” to “None of the time”. The responses are assigned to numerical signs, from 1 to 5 (rank). As items 4 and 12 indicate positive experiences (e.g., how often have you felt well rested) whereas items 3, 5, 6, 7, 8, 9, 10 and 11 indicate negative experiences (e.g., how often have you had an uneasy sleep), the latter were reversed such that a rank indicate more severe disruption. In the last page of the MOS questionnaire, we asked three open questions about perceived sleep patterns, quality and causes of sleep problems, if any.

The EORTC QLQ-C30 questionnaire consists of 30 items and is intended for a wide range of patients with cancer. It includes five functional scales, three symptom scales, single-item measures for additional symptoms, and two items assessing global health status/quality of life. Scoring of patient responses is done through a four-point Likert scale, which extends from 1 (not at all) to 4 (very much) and in some instances from 1 (very poor) to 7 (excellent).

### Psychometrics

Since the original MOS-SS questionnaire suggest up to seven different dimensions (amount of sleep plus six sub-scales), it is unclear how this instrument should be used in a routine clinical setting. A statistical ordinal factor analysis was therefore performed to evaluate if fewer and more intuitive readouts could be generated from the MOS-SS questionnaire.

To evaluate the psychometric properties of the readouts from the ordinal factor analysis, a Rasch analysis was performed on the new readouts as well as the individual items. In the Rasch analysis, responses to each question 3 to 12 of the MOS-SS were converted from the response values (ranks) to continuous values (odds), allowing the construction of an exponential model estimating how affected each patient is by a given response value to a question included in the MOS-SS and by the calculated sleep quality (SQ) and daytime sleepiness (DTS) factors (items).

$$P\left\{x_{ni}=x\right\}=\frac{e^{-\tau_{1i}-\tau_{2i}...-\tau_{xi}+x\left(\beta_{n}-\delta_{i}\right)}}{\sum_{x^{,}=0}^{m_{i}}e^{-\tau_{1i}-\tau_{2i}...-\tau_{xi}+x^{,}\left(\beta_{n}-\delta_{i}\right)}}$$

The model parameters are estimated from data and describes how well the data fits the model.

Finally, the results from the Rasch analysis is illustrated in person-item location distribution graphs where lower scores imply that a patient is less affected by that item compared to patients having higher scores.

### Serum biomarker tests

Plasma C-reactive protein (CRP) levels were analysed using Advia 1800 instrument with reagents from the same company (Siemens Healthcare, Erlangen, Germany).

Proteomic plasma biomarker analyses were initially performed on 17 patients, categorized into groups with either good (9 patients) or poor (8 patients) sleep using Human Cytokine/Chemokine/Growth Factor 45-Plex ProcartaPlex Panel 1 (ThermoFisher Scientific, Vienna, Austria).

From the proteomics 45-biomarker panel, 17 specific biomarkers (BDNF, Eotaxin, GM-CSF, GRO-a, HGF, IFN-g, IL-10, IL-12p40, IL-2, IL-5, IL-6, IL-7, LIF, MCP-1, PIGF-1, RANTES, and TNF-α) were selected. This selection included all biomarkers with statistically significant (*P* ​< ​0.10) difference in the plasma concentration of patients with cancer with “good” versus “poor” sleep quality at baseline, as well before and after three-month of oncological treatment.

The analysis of the selected 17 biomarkers was done on 54 patients using a custom ProcartaPlex 17-plex panel (ThermoFisher Scientific, Vienna, Austria) with multiplex fluorochrome technique (Luminex xMAP™ Technology, Austin, TX). Fluorescence intensities were analysed using a Bio-Plex 200 system with Bio-Plex Manager Software 5.0.

### Urine biomarker tests

Patients collected their first morning urine samples at home and brought them to the clinic on the first day of their treatment. Samples were also collected after three months. The three-month follow-up time correspond with the standard duration for most patients’ treatment cycles.

The concentrations of creatinine (glomerular filtration rate control), cortisol (day-time hormone), and melatonin (night-time hormone) were analysed using a calorimetric creatinine kit from R&D Systems (Minneapolis, MI, USA) a competitive ELISA kits from R&D Systems (Minneapolis, MI, USA) and Bühlman (Amherst, NH, USA). The measures were done using SoftMax® Pro 5 microplate reader.^24^^,^^25^ The levels of cortisol and melatonin were normalized to the levels of creatinine.

### Circadian gene expression tests

Based on their association to sleep, gene expression of 4 circadian clock genes (ARNTL, PER 1, PER 2 and PER3) were selected. RNA was extracted from Buffy coats generated from 10 mL EDTA blood and was stored in RNA later at −80°C until analysis. PureLink RNA Mini Kits was used with the protocol from the company in the isolation of the RNA. The concentration of the RNA was determined using The Qubit RNA BR (Broad-Range) Assay Kits and read by Qubit Fluorometer. cDNA synthesis was done from 100 ng/μL RNA using SuperScript VILO cDNA Synthesis Kit. Real-time PCR for the gene expression experiments was done using RT-PCR for TaqMan and Custom TaqMan Gene Expression Assay. All kits and instrument were from (ThermoFisher Scientific, Vienna, Austria). GAPH was used as reference gene for normalization of the analysed expression levels. All experiments were done according to the manufacturers’ instructions with Ct ​> ​37 as cut off level.

### Data analysis

Categorical data are presented as numbers, proportions and scores calculated from items with the same response dimension. Continuous data are presented as means and standard deviations or means with 95% confidence interval as indicated. Differences between two groups of continuous data were evaluated using students *t* test. The significance level was set to 5%, and a calculated *P*-value ≤ 0.05 is considered as statistically significant. For some of the variables Spearmans (ordinal data) or Pearson correlation (continuous data) was calculated to evaluate if there were any agreement in assessment or any linear association. A value of the correlation coefficient above 0.6–0.7 is of interest. However, correlation is not the same as causation. For the Rasch analysis RUMM2030™ 5.1 for Windows, © 1997–2010 Rumm Laboratory Pty Ltd, was used. More information about Rasch analysis can be found on https://rasch.org/

## Results

**Fig. 1:**
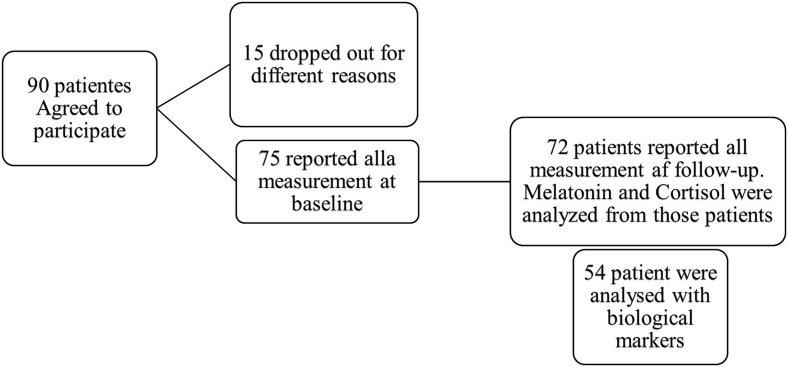
Patient flow chart. Illustration of the patient inclusion process and how many patients were used in the different types of analysis presented in this study. SQ, sleep quality; DTS, daytime sleepiness.

Seventy-five patients completed both the MOS-SS and EORTC QLQ-C30 questionnaires, and among them, 54 patients had complete data available for biomarker analysis (Table 1). Among the 21 patients who were not fully analyzed for all biomarkers, 3 passed away during the treatment period. For the remaining 18 patients, incomplete data were due to low RNA yield, assay failure, or other technical issues (Fig. 1).

**Table 1:** Demographic data of the 75 eligible patients

	*n* (%)	Systemic oncological treatments, alone or in combination
**Age, years** (min–max)	31–82	
Male, years (mean)	68	
Female, years (mean)	61	
**ECOG** PS		
0	46 (64)	
1	29 (36)	
**Sex**		
Female	28 (37)	
Male	47 (63)	
**Alcohol consumption**		
No	23 (31)	
Yes	52 (69)	
**Nausea experience before treatment**		
Pregnancy (only female)	8 (11)	
Travel nausea	7 (9)	
Not nausea at all	60 (80)	
**Occupation**		
Retired	49 (65)	
Worker	25 (33)	
Unemployed	1 (2)	
**Civil state**		
Married/partner	64 (85)	
Single	10 (13)	
Widower	1 (1)	
**Tobacco use**		
No	65 (87)	
Yes	10 (13)	
**BMI, kg/m^2^**		
Min	19	
Max	43	
Average	26	
**Tumour classification**		
Gastrointestinal cancer (pancreas, lever, oesophagus, rectal)	50 (67)	Folfox, Capecitabine, Fluorouracil, Bevacizumab, Flox, Gemox, Oxaliplatin, Gemcitabine, Irinotecan
Urothelial cancer (bladder, prostate)	11 (15)	Cisplatin, Carboplatin, Etoposid, Docetaxel, Mitomycin
Breast cancer	11 (15)	Trastuzumab, Paclitaxel, Epirubicin, Cyclophosphamide.
Others	3 (4)	Lomustine, Oncovin, Procarbazine.
**Comorbidity**		
Hypertension	9 (12)	
Diabetes	8 (11)	
Others	8 (11)	

ECOG PS, Eastern Cooperative Oncology Group performance status; BMI**,** body mass index.

### Two estimated sleep-dimensions suffice to explain responses to the MOS-SS

**Fig. 2:**
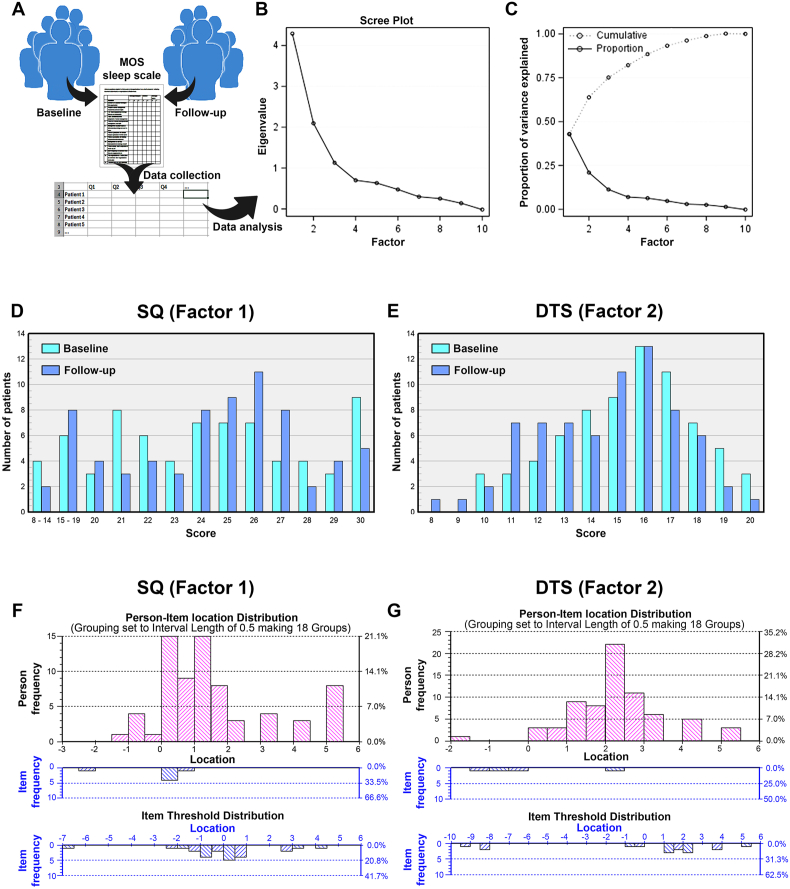
Ordinal factor and Rasch analyses identify SQ and DTS as the most important outputs from the MOS-SS scale. A) Cartoon illustrating the process of data collection and initial organization prior to further analysis. B) Scree plot of the Eigenvalue (mathematical significance requires an Eigenvalue > 2) of factors that may explain the variance in the data from patient-reported values to the MOS-SS. C) Proportion of the total variance in the data from patient-reported values to the MOS-SS explained by an increasing number of the factors identified in (B). D, E) Distribution of SQ (corresponding to Factor 1 in (B) and (C)) scores (D) and DTS (corresponding to Factor 2 in (B) and (C)) scores (E) among patients at the time om inclusion, prior to treatment onset (baseline) or following three months of oncological therapy (follow-up). F, G) Person-Item location distributions and Item Threshold Distributions derived from the Rasch analysis indicating the number (left axes) or frequency (right axes) of persons or items respectively found at each location within the SQ data (F) or DTS data (G). MOS-SS, medical outcomes study–sleep scale; SQ, sleep quality; DTS, daytime sleepiness.

Based on the responses to the items on the MOS-SS questionnaire collected before and after completed medical oncological therapy (Fig. 2A) two analyses were performed to investigate the potential co-variation of responses to items 3 to 12 of the scale, as well as their impact on the experience of sleep disruption in the patient.

First, we used an ordinal factor analysis of the responses from all 90 included patients at baseline to investigate the complexity of the variation in the responses from this patient group (Fig. 2B and C). Two factors were found to be significant for explaining the variance in responses (Eigenvalue > 2, Fig. 2B), and these two factors explained a combined 63% of the total variation (Fig. 2C), giving a reasonable coverage across the patient population. The first of these factors was found to consist of MOS-SS items 3, 4, 5, 7, 8, and 12, which are questions related to the sleep quality at night and was therefore abbreviated SQ, whereas the second consist of MOS-SS item 6, 9, 10 and 11, questions associated with daytime sleepiness and abbreviated DTS. The same pattern was found both at baseline and follow up. Table 2 shows the factor structure for MOS-SS data, and the contribution of each item to the two factors at baseline and follow-up. The SQ score was subsequently quantified as the sum of responses to MOS-SS items 3, 4, 5, 7, 8 and 12, with items 3, 5, 7 and 8 reversed, ranging from 6 (best) to 30 (worst). DTS is the sum of reversed items 6, 9, 10 and 11, ranging from 4 (best) to 20 (worst). To facilitate these calculations, an EXCEL-based tool was developed for easily deriving values for SQ and DTS based on assigned ranks to items 3–12 of the MOS-SS (Appendix 1). The distribution of the patient-reported scores to each item was similar at baseline and follow-up (Fig. 2D and E).

**Table 2:** Results from the ordinal factor analysis of MOS-SS items 3 to 12 at baseline (BL) and follow-up (FU).

Factor loadings			
Item	Question wording	SQ (BL/FU)	DTS (BL/FU)
3	How often during the past 4 weeks did you feel that your sleep was not quiet (moving restlessly, feeling tense, speaking, etc., while sleeping)?	88/76^a^	4
4	How often during the past 4 weeks did you get enough sleep to feel rested upon waking in the morning?	63/54^a^	45
5	How often during the past 4 weeks did you awaken short of breath or with a headache?	59/79^a^	−8
7	How often during the past 4 weeks did you have trouble falling asleep?	85/81^a^	13
8	How often during the past 4 weeks did you awaken during your sleep time and have trouble falling asleep again?	93/90^a^	12
12	How often during the past 4 weeks did you get the amount of sleep you needed?	82/79^a^	32
6	How often during the past 4 weeks did you feel drowsy or sleepy during the day?	39	66/57^a^
9	How often during the past 4 weeks did you have trouble staying awake during the day?	30	80/69^a^
10	How often during the past 4 weeks did you snore during your sleep?	−24	60/26^a^
11	How often during the past 4 weeks did you take naps (5 minutes or longer) during the day?	6	83/69^a^

a Indicated items included in the SQ or DTS factors. MOS-SS, medical outcomes study–sleep scale; SQ, sleep quality; DTS, daytime sleepiness.

Secondly, we evaluated the psychometric properties of SQ and DTS by performing a Rasch analysis on the calculated scores, as well as each of the contributing MOS-SS items, for each patient. In the Rash analysis, a total of 75 patients were included in the analysis. For the SQ factor 12 items were excluded due to incomplete records or extreme scores. For the DTS factor 7 items were excluded for the same reasons. The Person-item location distribution and the item threshold distribution (Fig. 2F and G) demonstrated that patients have a broader interpretation of the severity implied by changes in SQ compared to changes in DTS. Consequently, all items except item 4 were dislocated in the SQ score, whereas only one item was dislocated in the DTS score.

No significant change was observed in SQ from baseline to follow-up (Wilcoxon signed-rank test: S ​= ​−82.5, *P* ​= ​0.55). In contrast, a statistically significant change was observed in daytime sleepiness (DTS) scores (S ​= ​−300, *P* ​= ​0.025).

### Validation of SQ and DTS against other sleep disruption scales and measures

**Fig. 3:**
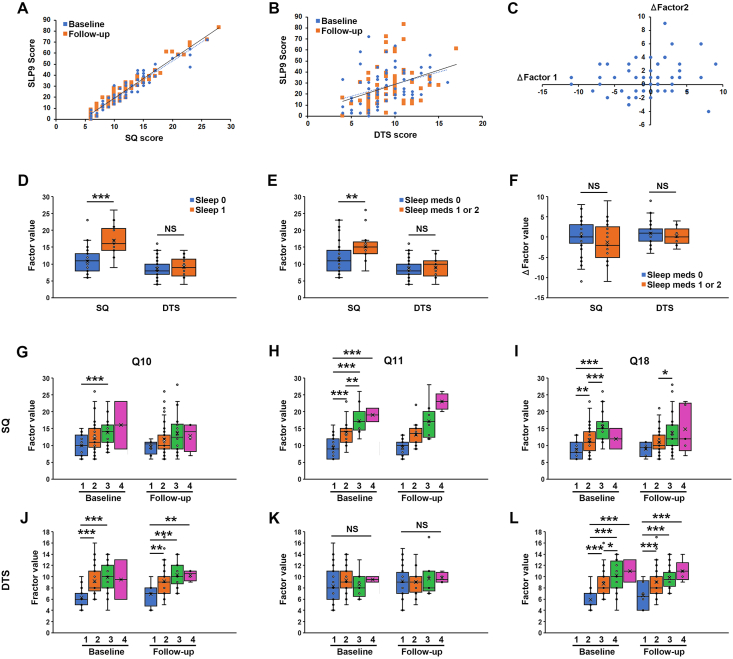
Correlation of SQ and DTS against other sleep scales and clinical data. A, B) Graph showing the association between SQ (A) or DTS (B) and the SLP9 sub-scale from the MOS Sleep Scale questionnaire at baseline (blue circles) and three months follow-up (orange squares). Each patient is represented by a blue and an orange data point in the graph. The correlation is significant at *P* ​< ​0.001 for both SQ (*R*^2^ ​= ​0.91 and 0.95) and DTS (*R*^2^ ​= ​0.13 and 0.13) and at both baseline and follow-up respectively. C) Graph showing the association between the change in SQ and change in DTS from baseline to follow-up. No statistically significant correlation was found (*P* ​> ​0.05, *R*^2^ ​= ​0.040). D, E) Box plots showing the average SQ or DTS at baseline for patients reporting poor sleep (Sleep 1, orange boxes, *n* ​= ​17) compared to good sleep (Sleep 0, blue boxes, *n* ​= ​56) in D or for patients taking sleep medication (orange boxes, *n* ​= ​17) compared to not taking sleep medication (blue boxes, *n* ​= ​53) in E. F) Box plots showing the average change in SQ or DTS between baseline and follow-up for patients taking sleep medication (orange boxes, *n* ​= ​17) compared to not taking sleep medication (blue boxes, *n* ​= ​53). NS: non-significant, ∗∗: *P* ​< ​0.01, ∗∗∗: *P* ​< ​0.001. G-L) Box plots showing average SQ (G–I) or DTS (J–L) in patients reporting a 1 (orange boxes, *n* ​= ​15, 35, 11 at baseline and *n* ​= ​8, 40, 6 at follow-up), 2 (blue boxes, *n* ​= ​37, 27, 40 at baseline and *n* ​= ​39, 16, 33 at follow-up), 3 (green boxes, *n* ​= ​19, 9, 20 at baseline and *n* ​= ​18, 9, 24 at follow-up), or 4 (purple boxes, *n* ​= ​2, 2, 2 at baseline and *n* ​= ​4, 4, 5 at follow-up) to questions 10 (G, J), 11 (H, K) or 18 (I, L) of the EORTC-QLQ30 instrument at either baseline or follow-up. NS: non-significant, ∗: *P* ​< ​0.05, ∗∗: *P* ​< ​0.01, ∗∗∗: *P* ​< ​0.001. MOS-SS, medical outcomes study–sleep scale; SQ, sleep quality; DTS, daytime sleepiness; EORTC-QLQ30, 30 item European organization for research and treatment of cancer-quality of life.

To validate the two new readouts from the MOS-SS, we statistically analysed the correlation between SQ and DTS values with the commonly used SLP9 score (from the MOS-SS) at baseline and follow-up. While both SQ and DTS correlated well with SLP9 at baseline and follow-up, the correlation was much stronger for SQ (Fig. 3A) compared to DTS (Fig. 3B). SQ and DTS had no statistically significant change globally from baseline to follow-up at the group level as evidenced by the correlation-lines for the baseline and follow-up datasets in Fig. A and B being near-identical. SQ and DTS scores, however, changed dramatically for some patients but these changes happened in both directions (e.g., sleep quality could deteriorate at follow-up in some patients but improve in others). The change in SQ values for individual patients did not correlate with the change in DTS values (Fig. 3C).

Patients who during the anamnesis either at baseline or follow-up reported that they were not sleeping well (in which case they would receive a score of one on a binary 0/1 scale) had significantly elevated values of SQ in the poor-sleep (category 1) patients compared to good-sleep (category 2) patients at baseline (Fig. 3D). DTS levels were, however, similar in both patient groups (Fig. 3D). In accordance with these findings, patients taking sleep medication either on an as-needed basis (category 1) or on a daily basis (category 2) also had significantly higher values of SQ compared to patients not taking sleep medication (category 0), whereas DTS was not changed among these patient groups (Fig. 3E). Both SQ and DTS, however, did not change (improve) significantly, neither in the sleep medicated or non-medicated patients, from baseline to follow-up (Fig. 3F).

We next investigated if SQ and DTS values correlated with responses to three questions from the EOATC-QLQ30 questionnaire related to sleep and sleepiness, namely question 10 (“have you needed rest”), question 11 (“have you had problems sleeping”) and question 18 (“have you been tired”). SQ increased robustly with increasing values to question 11 at both baseline and follow-up but exhibited weaker correlation and only at baseline to questions 10 and 18. DTS correlated robustly with increasing values of question 10 and 18 at both baseline and follow-up, but not at all with question 11.

### SQ or DTS does not correlate to levels of expected biomarkers of sleep disruption

**Fig. 4:**
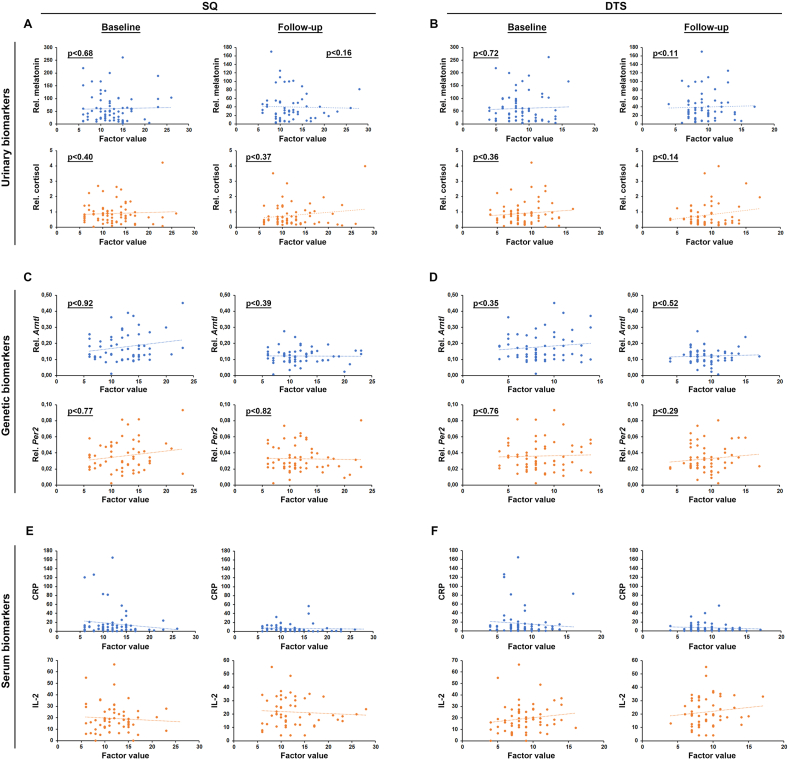
SQ and DTS are not correlated to levels of molecular biomarkers of sleep or circadian disruption in patients with cancer. A, B) Graphs showing the association between SQ (A) or DTS (B) levels and urinary relative levels of melatonin to creatinine (blue circles) or cortisol to creatinine (orange circles) at baseline or follow-up. Each patient is represented by a data point in each graph. C, D) Graphs showing the association between SQ (C) or DTS (D) levels and relative mRNA levels of *Bmal1* to *Gdpa* (blue circles) or *Per2* to *Gdpa* (orange circles) in blood leucocytes at baseline or follow-up. Each patient is represented by a data point in each graph. E, F) Graphs showing the association between SQ (E) or DTS (F) levels and serum levels of CRP (blue circles) or IL-2 (orange circles) at baseline or follow-up. Each patient is represented by a data point in each graph. There are no statistically significant correlations in any of the data sets (*P*-values indicated in each graph). SQ, sleep quality; DTS, daytime sleepiness; CRP, C-reactive protein; IL, interleukin.

We next investigated if higher or lower levels of some of the best described biomarkers of sleep disruption correlated with SQ or DTS scores in our patient cohort. The levels of melatonin or cortisol found in the first morning urine, the plasma levels of CRP or IL-2, or the gene expression level of circadian transcription factors Bmal1 or Per2 in leucocytes isolated from morning blood samples were measured. We expected that the following changes would correlate to poor sleep (e.g., higher SQ and/or DTS levels): Low levels of melatonin and/or high levels of cortisol in the morning urine; High levels of CRP and/or high levels of IL-2 in the plasma; High levels of Bmal1 and/or low levels of Per2 in leucocytes. None of the measured biomarkers, however, correlated with SQ nor DTS levels (*P* ​< ​0.11 – *P* ​< ​0.92), neither at baseline nor at follow-up (Fig. 4).

### Sleep disruption was independent of patient demography, treatment aggressiveness and type

**Fig. 5:**
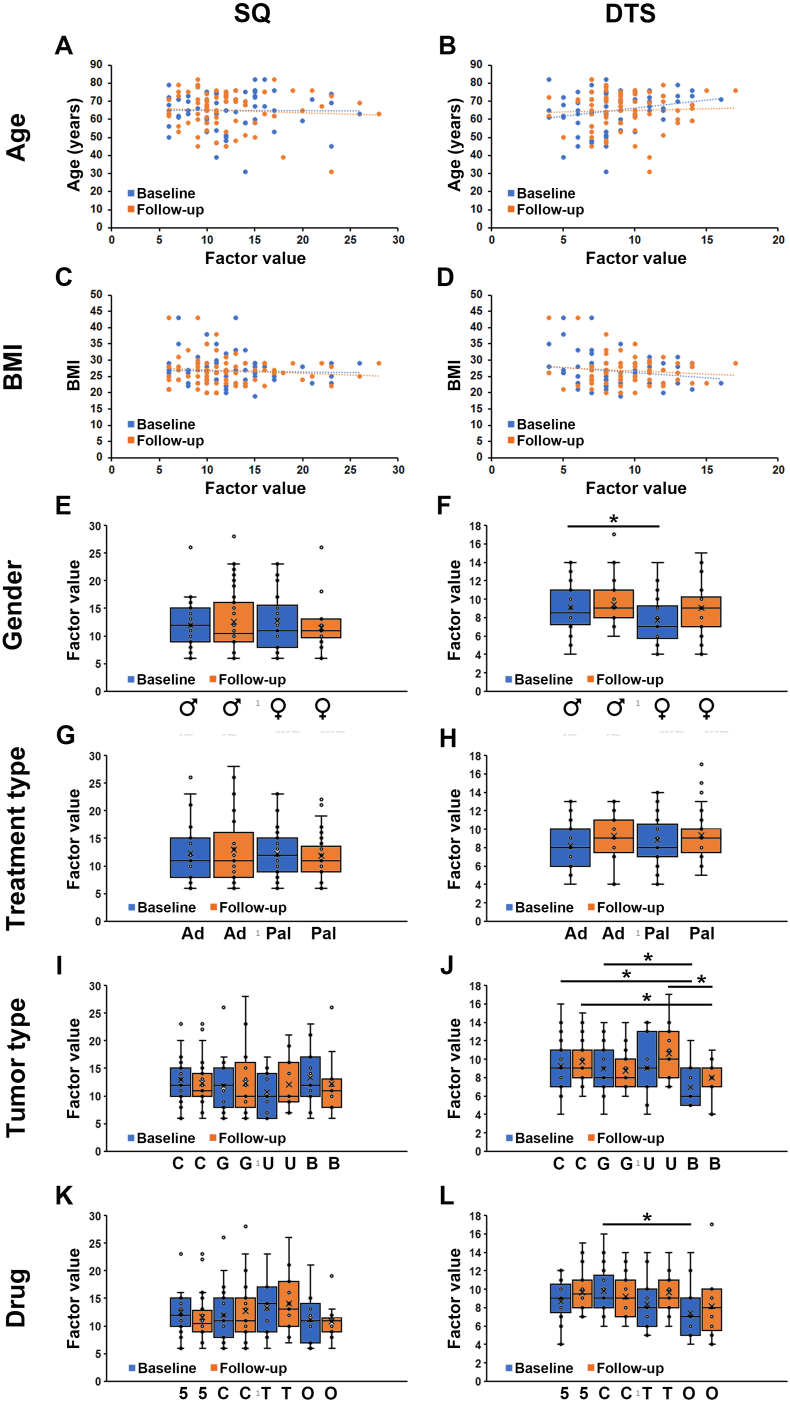
Correlation between SQ or DTS and demographic or clinical parameters in patients with cancer. A-D) Graphs showing the association between SQ (A, C) or DTS (B, D) and BMI (A, B) or Age (C, D) at baseline (blue circles) or follow-up (orange circles). Each patient is represented by a blue and an orange circle in each graph. There are no statistically significant correlations in any of the data sets (*P* ​> ​0.05). E, F), Box plots showing the average SQ (E) or DTS (F) levels for patients identifying as male (*n* ​= ​44) or female (*n* ​= ​26) at baseline (blue boxes) or follow-up (orange boxes). ∗: *P* ​< ​0.05. G, H), Box plots showing the average SQ (G) or DTS (H) levels for patients receiving adjuvant (Ad, *n* ​= ​25) or palliative (Pal, *n* ​= ​45) therapy at baseline (blue boxes) or follow-up (orange boxes). I, J), Box plots showing the average SQ (I) or DTS (J) levels for patients diagnosed with colorectal (C, *n* ​= ​31), gastric (G, *n* ​= ​19), urological (U, *n* ​= ​11), or breast (B, *n* ​= ​11) cancer at baseline (blue boxes) or follow-up (orange boxes). ∗: *P* ​< ​0.05. K, L), Box plots showing the average SQ (K) or DTS (L) levels for patients treated with therapies containing 5-FU (5, *n* ​= ​25), any -citabine (C, *n* ​= ​25), any taxane (T, *n* ​= ​11), or other (O, *n* ​= ​13) medical therapy at baseline (blue boxes) or follow-up (orange boxes). ∗: *P* ​< ​0.05. SQ, sleep quality; DTS, daytime sleepiness; BMI**,** body mass index.

SQ and DTS were similar across all age-groups (Fig. 5A, B) and across patients with different body mass index (BMI) (Fig. 5C, D) both at baseline and follow-up. SQ was also similar for both genders (Fig. 5E), but DTS was significantly lower for women compared to men at baseline (Fig. 5F). As adjuvant therapy was expected to be more toxic than palliative therapy and therefore potentially associated with more treatment-associated side effects, which could include sleep-disruption, it was investigated if SQ or DTS scores differed among patients given adjuvant or palliative therapy. Surprisingly, both SQ and DTS were similar in patients within these two treatment regimens at follow-up (Fig. 5G, H), in line with our observation that SQ or DTS levels did not change at follow-up compared to baseline in the patient population as a whole. Patients with breast cancer were found to have a significantly lower DTS level compared to both patients with colorectal cancer and other gastrointestinal cancers at baseline and compared to patients with colorectal cancer and urothelial cancer at follow-up (Fig. 5I and J respectively). The type of medical treatment given (flurouracil-, citabine-, or taxane-containing therapy, or other) had no impact on sleep disruption measured using SQ or DTS (Fig. 5K, L).

## Discussion

Poor sleep is an important care-related parameter affecting quality of life, treatment outcomes and survival in patients with cancer.^26^ Sleep disruption, whether diagnosed by an objective device or subjective self-reported instrument, is however, closely associated with shortened overall survival in patients with advanced colorectal^20^ and head and neck cancer.^27^ Patients affected by sleep disruption are also diagnosed with more advanced stages of cancer compared to those with a healthy sleep rhythm.^28^ In spite of its clinical importance, sleep disruption is often overlooked in cancer care due to the fact that other, more severe symptoms demand the focus of the nurses.^29^ The problem is aggravated by a lack of intuitive, fast, and easy to use tools for diagnosing sleep disruption in patients with cancer. As a consequence, effective sleep-correcting measures are currently underused within clinical oncology.^30^, ^31^, ^32^

Here we took a mathematical and psychometric approach to investigate the co-variation and significance of answers to the popular MOS-SS questionnaire. The outcome is a new, fast, and simple tool to identify sleep disruption in patients with cancer (Appendix 1). Specifically, we found that two calculated scores (SQ and DTS) accurately captured the information from the questionnaire, and that these two scores sufficed to understand the variation in sleep disruption experienced by the patients. Focusing only on the SQ and DTS scores greatly simplifies the interpretation of the MOS-SS compared to the six previously suggested sub-scales from this questionnaire, allowing faster, less confusing, clearer and more intuitive diagnostic readouts to be obtained from the MOS-SS, thereby facilitating implementation of this instrument in routine clinical practice. Exploiting the simple “SQ and DTS calculation tool” provided in Appendix 1, it takes only a minute or two to understand the degree of sleep disruption a patient is suffering from, and whether this mainly affects night-time sleep or daytime sleepiness of the patient.

To further investigate the importance of the SQ and DTS factors, we performed a Rasch analysis of both these factors as well as the included MOS-SS items. The items of the MOS-SS are corresponding to separate sleep-related issues and can, from a statistical point of view, as ordered categorical data imply that the distance between response categories (e.g., a response of 3 or 4) does not signify the same distance in experienced severity of that issue for different patients. For one responder, a change of one step on the scale (e.g., from 4 to 3) may correspond to a large improvement for that issue, while a two-step change (e.g., from 4 to 2) for another patient might correspond to only a moderate improvement. This makes it difficult to evaluate what each response actually means for the patient by simply looking at the data itself. The idea behind the Rasch analysis is to use odds (that converts categorical data to continuous data) to estimate the experienced severity that each score to a MOS-SS item imply for a given patient.^33^ Using this analysis, we found that all but one item in the MOS-SS were dislocated in the SQ factor, which suggests that there might be too many response categories (e.g., 1 through 5, where possibly 1 through 3 would suffice) for these items in the MOS-SS. This was also evident from the broader person-item location distribution for the SQ factor compared to the DTS factor. In other words, a certain SQ value could mean different things (e.g., either a poor or very poor experienced sleep quality), and a change of 3 in this factor could be seen as more or less significant, for different patients. The DTS factor, on the other hand, was more congruent among the patients in this study. This result exemplifies the difficulties in accurately measuring patients with cancer experience of their sleep quality. The complexity of the issue is cooperated by the current controversy of whether objective or subjective measurements of sleep disruption are the most relevant, and to what extend sleep disruption is associated with molecular biomarkers (recently reviewed in^10^).

Looking at the absolute values and changes in values between baseline and follow-up, SQ and DTS were found to be independent of each other. This was a surprising finding as we expected based on a study with heart failure patients that poor sleep would have been associated with increased daytime sleepiness,^34^ but such a relationship was not seen in this study. Similar to our findings, Lund et al. also did not see a relationship between poor sleep quality and excessive daytime sleepiness in young college students,^35^ suggesting that this relationship is not universal and may be dependent on age- and health context. We hypothesize that the uncoupling of these two factors in patients with cancer may be due to disease- or therapy-related factors such circulating factors causing sleepiness during the day (e.g., fatigue) even in patients with adequate sleep at night. Inflammatory cytokines such as TNF-a and IL-6 have been shown to have such sleepiness-inducing effects.^36^, ^37^, ^38^ We were not able to find any correlation between these factors and the DTS score in this study, suggesting that other inflammatory or other circulating factors not included in this study may also have a similar role. The lack of impact of biomarkers tested in this study on sleep disruption in the patients could also potentially be explained by the psychosocial stress experienced by the patient due to the cancer diagnosis and -treatment. Psychosocial stress is a common, multifaceted, inflammation-modulating factor that impacts patients differently depending on stress duration and degree of support in the patient's environment.^39^ It is therefore possible that psychosocial stress makes the biomarker profile broader and more complex masking the role of single sleep biomarkers in the context of patients with cancer.

Neither SQ nor DTS was found to change over time in the patient group as a whole, compared to the start of oncological medical therapy. This suggests that oncological therapy does not influence overall sleep quality or day-time sleepiness of the patients with cancer as a group. Furthermore, SQ or DTS did not correlate with molecular biomarkers of circadian disruption such as the melatonin or cortisol levels in morning urine, inflammatory cytokines in the blood or circadian transcription factors Bmal1 or Per2 in leucocytes. Also, this finding was surprising as these biomarkers were chosen because they have been shown to be associated with sleep disruption in the past.^6^^,^^40^^,^^41^ These and other previous studies, however, were done on a healthy population (i.e. people included as “healthy research subjects” without known disease), or more defined populations of patients with cancer. Our results suggest that such commonly used urinary, serum or gene-expression biomarkers of sleep disruption are not broadly applicable to a diverse population of patients with cancer undergoing treatment at oncology clinics, possibly due to the influence of their varying severity of disease, co-morbidities, and side-effects from therapy. Other studies have similarly found a lack of correlation between experienced sleep quality and sleep-related biomarkers in patients with cancer,^10^^,^^18^^,^^42^ lending support to the hypothesis that sleep disruption can only be diagnosed using subjective instruments in this patient group.

Interestingly, we can in this study report that female patients with cancer experience less daytime sleepiness, reflected by a low DTS score, compared to male, since patients with breast cancer in general having lower DTS scores than colorectal or gastrointestinal patients with cancer. Breast patients with cancer were commonly receiving adjuvant treatment while the patients with gastrointestinal cancer were in the palliative setting, which could influence DTS. The lower DTS was however observed both before and after medical therapy. The fact that female patients with cancer are less sleepy during the day compared to male patients with cancer, is likely not a general aspect associated with female gender as young female college students were found to have higher DTS than males,^43^ but is suggested from other studies on sleep in patients with male- or female cancers.^44^^,^^45^ The reasons underlying this positive quality-of-life aspect in patients with cancer, have not been investigated previously but deserves attention also in future studies.

In this study we were able to correlate SQ and DTS scores to an aggregated feeling of good/poor sleep derived from the anamnesis as well as EORTC-QLQ questions related to daytime sleepiness. We found that 15 of 17 patients reporting poor sleep during the anamnesis had an SQ score of 14 or higher whereas 47 of 56 patients reporting good sleep had an SQ score lower than 14. Similarly, 18 of 22 patients indicating more pronounced need to rest or 23 of 29 indicating more pronounced tiredness (3 or 4 in the EORTC QLQ-C30) had a DTS score of 9 or higher whereas 33 of 47 patients indicating less pronounced need to rest or 28 of 39 patients less pronounced tiredness (1 or 2 in the EORTC QLQ-C30) had a DTS score of less than 9. We therefore suggest that an SQ cut-off of 14 and a DTS cut-off of 9, calculated using the tool in Appendix 1, may hold promise for diagnosing sleep disruption in patients with cancer within clinical routine in the future.

Both SQ and DTS did not change significantly in patients given sleep medication to correct their poor sleep quality. Similar to other studies showing minimal or no effect of medical interventions to correct sleep quality in patients with cancer,^10^ this finding suggests that other interventions are required to handle poor sleep quality in this patient group. The role of such alternative sleep correcting interventions was recently reviewed,^10^ but previous studies lack a personalized approach – i.e. to only offer sleep correcting interventions to patients affected by sleep disruption within the oncology setting. Future studies should focus on evaluating, also in patients at oncology clinics, the potential effects of non-medical sleep correcting interventions in a personalized manner.

In the healthy population sleep quality and circadian rhythms are partially disrupted as we grow older and with increasing BMI.^38^ In addition, other demographic parameters such as sex, or disease-associated parameters such as whether a patient receives aggressive adjuvant therapy compared to less aggressive palliative therapy, may affect sleep or sleepiness. Here we found that in patients with cancer SQ and DTS were similar across all age groups and BMI levels, suggesting that patients with cancer may have a generalized disruption in SQ or DTS that is stronger than the effects of age and BMI in a healthy population, thereby masking that correlation in our data set. Surprisingly, we also did not find a more severely affected SQ or DTS among patients receiving adjuvant compared to palliative therapy. We hypothesize that this may be due to a similar degree of improved and reduced SQ and DTS in patients having a net benefit compared to toxicity from either type of therapy thereby masking an overall effect on the group level. Future studies comparing the change in SQ and DTS in patients having an effect from the medical therapy compared to those having mainly toxic side-effects from the therapy are required to understand if medical oncological therapy can be associated with both sleep-restoring and sleep-deteriorating effects in the patients.

### Strengths and limitations of the study

This single-center pilot study involved a small and diverse group of patients with cancer, and focused primarily on detecting sleep problems during anticancer treatment. Patients included both those undergoing adjuvant and palliative therapy for various indications and had a varying health status and broad demographic profile. The “real life” character of the study is a strength as it captures the real complexity and variation found in oncology clinics. However, there are also several limitations to consider. The small size of the study, coupled to issues that meant we could not measure all biomarkers for approximately 25% of the patients, did not allow an identification of sleep-quality related biomarkers in this study. Furthermore, urine samples may not be first morning urine, as collection was patient-managed, causing potential result variations. Our study was also not powered to allow complete stratification among patient sub-groups, drug treatments or disease severity although an attempt to do so by combining similar types of patients and drugs was made. The study was a single center study with inclusion during one year, limiting the socioeconomic and demographic context of the study. Hence, results may not generalize to all patients with cancer, indicating a need for further studies.

Future studies should examine how medical therapies impact sleep, differentiating between therapeutic benefits and toxic side effects. Additionally, non-medical interventions like physical exercise and cognitive behavioural therapy, which have improved sleep in other groups, should be explored.^46^, ^47^, ^48^

## Conclusions

Here we show that two simple factors derived from the MOS Sleep Scale – SQ and DTS accurately captures cancer patient sleep quality and daytime sleepiness respectively and validate the use of these factors for detecting these aspects of sleep disruption in the patients. We propose a fast and simple tool to identify patients with cancer with sleep disturbance (Appendix 1) for potential use in clinical practise following further clinical validation. We further show that SQ and DTS are not, within this small pilot study, affected by medical oncological therapy regardless of type or aggressiveness, and that, in patients with cancer, both SQ and DTS does not change with increasing age or BMI. Furthermore, SQ and DTS did not correlate with molecular biomarkers of sleep or circadian disruption such as melatonin, cortisol, inflammatory cytokines or circadian transcription factors, in this patient group. Female patients with cancer, however, did show a markedly lower DTS level compared to male patients, which may have implications on adherence to and/or effectiveness of care interventions and activities scheduled during the day, a subject that deserves further investigation in future studies.

## CRediT authorship contribution statement

DO: Conceptualization, Methodology, Investigation, Data Curation, Writing – Original Draft. BÅA: Conceptualization, Methodology, Investigation, Formal Analysis, Writing – Review & Editing. FL: Conceptualization, Methodology, Supervision, Writing – Review & Editing, Funding Acquisition. LJ: Conceptualization, Methodology, Investigation, Formal Analysis, Writing – Review & Editing. MN: Formal Analysis, Visualization, Writing – Original Draft authors have read and approved the final manuscript. All authors have read and approved the final manuscript.

## Ethics statement

The study was approved by the Regional Ethical Review Board in Linköping, Sweden (Approval No. Dnr:2016/379-31e) and was conducted in accordance with the 1964 Helsinki Declaration and its later amendments or comparable ethical standards. All participants provided written informed consent.

## Data availability statement

The data that support the findings of this study are available from the corresponding author, DO, upon reasonable request.

## Declaration of generative AI and AI-assisted technologies in the writing process

No AI tools/services were used during the preparation of this work.

## Funding

The study was supported by the Foundation for Clinical Cancer Research in Jönköping and Futurum Academy for Health and Care (Grant No. 866371), Swedish Cancer Research Association (Grant No. Cancerfonden, Grant No. 23 2954 Pj 01 H). The funders had no role in considering the study design or in the collection, analysis, interpretation of data, writing of the report, or decision to submit the article for publication.

## Declaration of competing interest

The authors declare that there are no conflicts of interest.
